# Construction Notes

**Published:** 1905-08-26

**Authors:** 


### CONSTRUCTION NOTES.

A new Infirmary Annexe has been opened at the Parkside County Lunatic Asylum. A hand-drawn ambulance conveys the patients from the Asylum to the Infirmary buildings. The accommodation is for 206 patients, and the cost was £60,000.

The Great Ormond Street Hospital for Children has received 50 guineas from the Grocers' Company and £30 from both the Fishmongers' Company and the Merchant Taylors' Company. The Homœopathic Hospital has received £250 through the will of the late Miss C. Petter of Bournemouth.

